# Phenotypic Evolutionary Models in Stem Cell Biology: Replacement, Quiescence, and Variability

**DOI:** 10.1371/journal.pone.0001591

**Published:** 2008-02-13

**Authors:** Marc Mangel, Michael B. Bonsall

**Affiliations:** 1 Center for Biomolecular Science and Engineering, Department of Applied Mathematics and Statistics, University of California Santa Cruz, Santa Cruz, California, United States or America; 2 Mathematical Ecology Research Group, Department of Zoology, University of Oxford, Oxford, United Kingdom; University of Sheffield, United Kingdom

## Abstract

Phenotypic evolutionary models have been used with great success in many areas of biology, but thus far have not been applied to the study of stem cells except for investigations of cancer. We develop a framework that allows such modeling techniques to be applied to stem cells more generally. The fundamental modeling structure is the stochastic kinetics of stem cells in their niche and of transit amplifying and fully differentiated cells elsewhere in the organism, with positive and negative feedback. This formulation allows graded signals to be turned into all or nothing responses, and shows the importance of looking beyond the niche for understanding how stem cells behave. Using the deterministic version of this framework, we show how competition between different stem cell lines can be analyzed, and under what circumstances stem cells in a niche will be replaced by other stem cells with different phenotypic characteristics. Using the stochastic version of our framework and state dependent life history theory, we show that the optimal behavior of a focal stem cell will involve long periods of quiescence and that a population of identical stem cells will show great variability in the times at which activity occurs; we compare our results with classic ones on quiescence and variability in the hematopoietic system.

## Introduction

The current enthusiasm for stem cells (both adult and embryonic) [1], and their role in regenerative medicine is based on the assumption that we can remove stem cells from their natural habitat, propagate them in culture, transplant them into a foreign environment and assume that the transplanted cells will do as we wish or that we can manipulate them *in vivo* with desired results [2]. For example, stem cells can differentiate into cell lineages that are different from the tissue in which they originate and adult stem cells can migrate to sites of injury [3]. Harold Varmus has said that “It is not unrealistic to say that [stem cell] research has the potential to revolutionize the practice of medicine and improve the quality and length of life” (pg. 513 in [4]). But long before that, Theodosius Dobzhansky said that us “nothing makes sense in biology except in the light of evolution” [5, pg 449].

There may be enormous differences between what stem cells do in their original tissue and what they can and will do when put into culture or when transplanted to a new location [6]. Raff [7] noted that “perhaps the greatest challenge in stem cell biology is to uncover the…mechanisms that determine whether a daughter of a stem cell division self-renews or commits to a particular pathway of differentiation. Cracking this problem for the adult mammalian stem cells of interest will be a crucial step for both developmental biology and cell therapy.” Solutions in this area of stem-cell biology will require all sorts of different biology, and innovative ideas. Elsewhere [8] we have argued that the time is right for the development of evolutionary theory about stem cells and the microenvironment (their niche) that maintains them.

It is generally agreed that a focal stem cell in its niche is influenced by signals (both positive and negative feedback) from the other stem cells in the niche, the support cells in the niche, and from more differentiated cells throughout the body. How exactly to model this is unclear, with the consequence that most models of stem cells focus on a single stem cell (or a population of identically behaving stem cells) in a single niche. We show how to make operational the recommendation in [9] that the next advances will combine probability-based events and mechanistic parameters as the best approximation to actual phenomena. Our models are rooted in evolution by natural selection, which is ultimately the mechanism for all of biology.

Theoretical approaches to stem cell biology have usually involved typological thinking [10], although there are examples of population thinking [11]. Typically, one assumes that all stem cells behave in a similar manner and writes either a set of deterministic differential equations or a probabilistic master equation [12] to characterize the dynamics of stem cells, but ignores natural selection. What is now required is a kinetic theory of stem cell dynamics that accounts for variation and natural selection [13]. We offer the first such theory here. We also show how the biological version of the Heisenberg uncertainty principle [14], [15] can be made operational to show how manipulations may alter the behavior and progeny of stem cells. To achieve our goals, we use a somewhat stylized model, motivated by the hematopoietic system. There is much to be learned from such stylized models, in systems biology [16], ecology [17], [18], and evolution [19]–[21].

September 2007 was the 50th anniversary of the publication of the first successful demonstration of the intravenous infusion of bone marrow in patients who had experienced radiation or chemotherapy that destroyed bone marrow [22], [23]. In a subsequent classic study of the hematopoietic stem cell system [24], stem cell colony forming units showed enormous epigenetic variation, leading to highly overdispersed numbers of descendant cells in individual colonies in spleens of experimental animals. We will show how to interpret this result in terms of variability in the cell cycle [25]; this begs the question of why such variation has not been eliminated, or alternatively how natural selection maintains such variation.

In its niche, a stem cell responds to its local environment (signals received from the support cells of and the other stem cells in the niche) and its global environment (signals received from more differentiated cells) but we assume that the behavior of the focal stem cell does not affect the behavior of the other stem or more fully differentiated cells. We are thus able to avoid, at least initially, game theoretical aspects of stem cell biology; ultimately they must be considered. Even with this simplified framework, we are able to understand how natural selection will act on competition between stem cells with different phenotypic characteristics (explained below) and how natural selection can lead to both quiescence and great variation in the behavior of stem cells.

### Overview of the Models

Stem cells have the ability to both self-renew and to differentiate. The niche of the stem cell is the local microenvironment that supports the maintenance, renewal, and differentiation of stem cells. In the case of the villi of the small intestine or bone marrow it is easy to conceive of the niche as a defined physical environment. In other cases, such as the epidermis, the niche will be determined by the spatial extent of signaling molecules. Signals and feedback controls (both negative and positive) affect the pattern of behavior of the stem cells in their niche. These signals include cell-autonomous promotors (such as *piwi*); self-feedback, autocrine and paracrine influences; hormones, growth factors, cytokines, and short-range cell to cell signalling pathways (e.g. Notch, Hedgehog, Wnt); hormones from remote sources; positional cues from chemical gradients as in classical Turing systems [26]; and adhesion [27]. Thus, stem cells are not simply single input-output systems, but are complex, nonlinear, multiple input-output systems [28].

**Figure 1:**
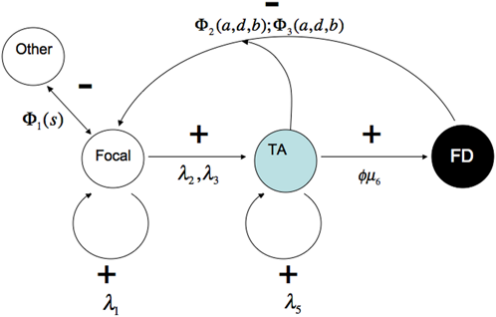
The feedback network affecting a stem cell in its niche. Here stem cells are clear circles, transit amplifying cells colored circles, and fully differentiated cells in black. A focal stem cell has positive feedback (+) on itself but shares inhibition and negative feedback (−) with other stem cells. Transit amplifying cells receive positive feedback from stem cells and exert positive feedback on fully differentiated cells. Both transit amplifying and fully differentiated cells exhibit negative feedback on stem cells. The symbols that characterize these processes are explained in the Materials and Methods.

The simplest population biology that one can imagine is to focus on the number of stem *S*(*t*), transit amplifying progenitor *A*(*t*), and fully differentiated *D*(*t*) cells at time *t* associated with the stem cells in the niche being modeled. In doing so, for simplicity we compress the differentiation stages of a transit amplifying cell, but they could be unpacked should one wish to do so. In addition, there will be other transit amplifying and fully differentiated cells, produced by other stem cells, that create an additional signaling background. We denote those cells by *B*. For simplicity we consider only one kind of progenitor cell and only one kind of differentiated cell, but extensions are clear and obvious. The positive and negative feedback links affecting stem cell fate are illustrated in Figure 1.

### The transitions of stem, transit amplifying, and fully differentiated cells

Most simply, stem cells divide symmetrically – doubling and producing two stem cells, divide asymmetrically – doubling and producing one stem cell and one cell that will be a progenitor of a fully differentiated cell, undergo apoptosis (to maintain an error-free genome [29], [30]) or migrate out of the niche [31]. Almost all of the literature focuses on whether stem cells will divide symmetrically or asymmetrically.

In fact, there are many more possible transitions of stem cells and their progeny. We use the following indexing system for transitions of stem, transit amplifying and fully differentiated cells: 1) a stem cell divides symmetrically, 2) a stem cell divides asymmetrically, 3) a stem cell divides and differentiates symmetrically producing two transit amplifying cells, 4) a stem cell under goes apoptosis or migrates out of the niche, 5) a transit amplifying cell divides, 6) a transit amplifying cell undergoes apoptosis, 7) a transit amplifying cell fully differentiates, 8) a fully differentiated cell undergoes apoptosis. To fully understand the evolutionary ecology of stem cells we need to consider the entire range of possibilities of transitions and both positive and negative feedback. This is implicit in [32]. In **Materials and Methods** we describe how to model these transitions, implement the model and associate a system of ordinary differential equations with the conditional means of the stochastic numbers of stem, transit amplifying, and fully differentiated cells.

### Trade offs

The transitions of stem, transit amplifying, and differentiated cells are characterized by a number of parameters. Biological realities will constrain some choices for parameter values. For example, we may anticipate that the probability that all stem cells in the niche either undergo apoptosis or migrate out of the niche is not too high since if it were the system could not persist. That is, if the niche empties (either through apoptosis or migration) it has essentially gone extinct. Similarly, in most situations, the population of transit amplifying cells is much more active than the population of stem cells (with consequence that the number of amplifying cells will thus be larger than the number of stem cells) and we require parameter values that make this so. We also require that when there is just one stem cell in the niche, the numbers of stem cells increase.

In addition, we may expect there to exist links and trade-offs between the different parameters. For example, since symmetric renewal, asymmetric renewal and symmetric differentiation involve many of the same signals and processes, we may expect that their rates are correlated. Cells that undergo high rates of replication are more likely to have errors in them and thus are likely to undergo apoptosis. When transit amplifying cells disappear at a higher rate because of replication error, fewer of them will become fully differentiated cells and this too can be captured by a trade-off.

In summary, one may conceive of the parameter set describing the transitions of the stochastic kinetics as the phenotype of a stem cell and its descendants, or if all of the stem cells in a niche are identical descendants of a single cell, then these parameters are the phenotype of the stem cell clone. These trade-offs are fully described in **Materials and Methods**.

### The focal stem cell in the niche

To understand fully how natural selection acts on a stem cell, we need to consider the fitness of a focal stem cell. Stem cells (and transit amplifying cells) do not by themselves achieve fitness. Rather, they support the organism – which can be viewed as an organized collection of fully differentiated cells. Fitness of the focal stem cell depends upon what it does, what the other stem cells in the niche do, and how many transit amplifying and differentiated cells are present. We thus define a fitness function *F*(*y*, *s*, *a*, *d*) to be the maximum expected accumulated fitness through differentiated cells, given that the focal stem cell has accumulated *y* resources towards the next division, that there are *s* stem cells in the niche and that there are *a* and *d* transit amplifying and differentiated cells sending signals back to the focal stem cell. State dependent life history theory, as implemented through stochastic dynamic programming [33]–[36], allows us to compute the fitness-maximizing responses of the focal stem cell.

## Results

A variety of parameters are used in this section; they are explained in Table 1 and constitute the phenotype of the stem cell and its descendants. For modeling, it is generally easier to work with the rates of cell cycles (generically, λ) rather than cell cycle times (generically *T*).

**Table 1:** Parameters, Their Interpretations, and Values.

Parameter	Interpretation	Value
*K*	Parameter of feedback inhibition of stem cells on each other (Larger K implies less inhibition)	10
λ_1_	Cell cycle rate for symmetric renewal	0.10
λ_2_	Cell cycle rate for asymmetric renewal	0.20
λ_3_	Cell cycle rate for symmetric differentiation	0.01
μ_40_	λ_1_ independent per capita rate of stem cell migration and apoptosis	0.01
μ_41_	λ_1_ dependent per capita rate of stem cell migration and apoptosis	0.1
λ_5_	Per capita rate of transit cell amplification	0.05
μ_60_	λ_1_ independent rate of disappearance of transit amplifying cells	0.05
μ_61_	λ_1_ dependent rate of disappearance of transit amplifying cells	0.5
φ_0_	λ_1_ independent probability that a transit amplifying cell differentiates	0.9
φ_1_	λ_1_ dependent reduction in φ	1.0
μ_8_	Mortality rate of fully differentiated cells	.04
ε	Parameter for feedback inhibition from amplifying and differentiated cells	0.001
γ	Parameter for feedback inhibition from amplifying and differentiated cells	0.01
*B*	Background level of transit amplifying and fully differentiated cells	100
*dt*	Time step for iterating the equations	0.01
*y_d_*	Threshold level of resources needed for differentiation	5.5
γ*_c_*	Sensitivity of error correction to resources	3.0

### Another look at Till et al (1964)

**Figure 2:**
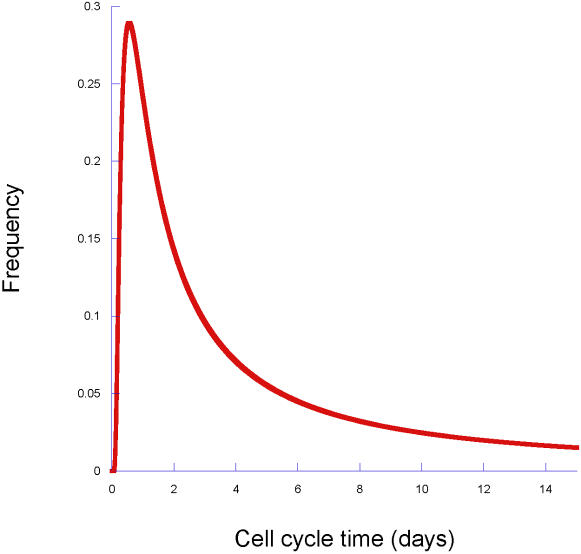
The distribution of cell cycle times inferred from the overdispersed data of Till et al. This should be compared to Figure 6.

We begin with another look at Till et al [24]. Their data lead to a frequency distribution for cell cycle times that is shown in Figure 2. We are again led to ask how natural selection maintains such a broad and slowly declining distribution.

### A special case of the differential equations

If the rate of symmetric differentiation is 0, we can solve for the steady state levels of stem and differentiated cells explicitly and easily obtain that of the amplifying cells numerically. In particular, the steady state number of stem cells is
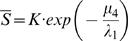
(1)which shows that the steady state number of stem cells increases with increasing values of λ_1_ and with decreasing values of μ_4_ (Table 1), both of which accord with intuition. If the steady state number of transit amplifying cells were known, the steady state number of differentiated cells is
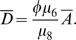
(2)Thus, by adjustment of any of φ, μ_6_ or μ_8_ (Table 1), natural selection can lead to different numbers of fully differentiated cells without changing the fundamental structure of the system of stem, transit amplifying and differentiated cells. That is, we can create a ratio of fully differentiated to stem cell that ranges from the observed 3∶1 to 2–5,000∶1 through the evolution of phenotypic characteristics.

We obtain a single nonlinear equation for the steady state level of transit amplifying cells
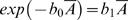
(3)where 
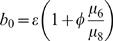
 and 
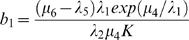
.

This special case also serves as a check of our numerical methods.

### Qualitative properties of the stochastic system

As described in the **Materials and Methods**, under very general conditions, *S* = 0 is dynamically unstable and has maximum value *K* (determined by feedback inhibition). Thus, the solution of the set of differential equations will move from *S* = 1 (the minimum non-zero value of the number of stem cells in the niche) towards *K*, determined by the balance of μ_4_ and λ_1_. In the full stochastic simulation, all the same properties hold, as can be seen from the equations characterizing the transition rates. One important consequence of *S* = 0 being dynamically unstable is that when a niche goes extinct, it will do so quickly [25]. Thus, we expect extant clones of stem cells to have much longer lifetimes than those of clones heading for extinction. Similarly, the number of transit amplifying cells is determined by the balance of μ_6_−λ_5_ and φ. The number of fully differentiated cells is determined by μ_8_, φ, and μ_6_ – thus a variety of simple mechanisms can create tissues with different structures.

### Quantitative results

**Figure 3:**
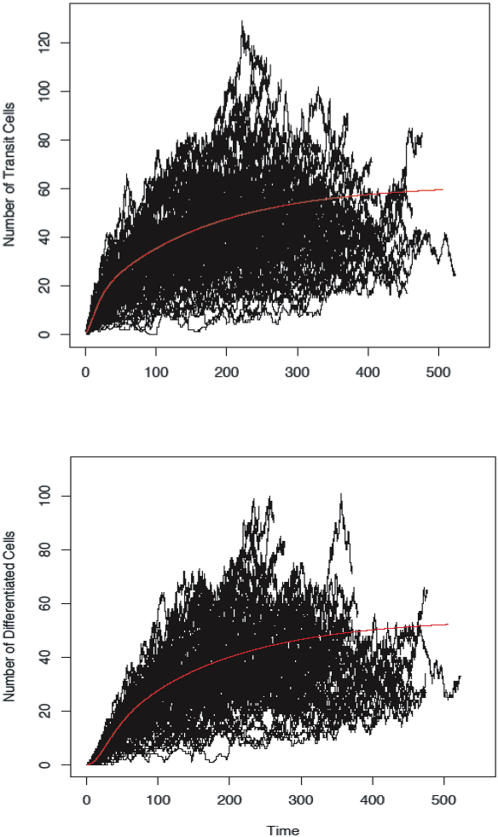
The stochastic trajectories for 100 simulations of the stem-transit amplifying-differentiated system described in the text and the solution of the differential equations 25–27 (red line).

**Figure 4:**
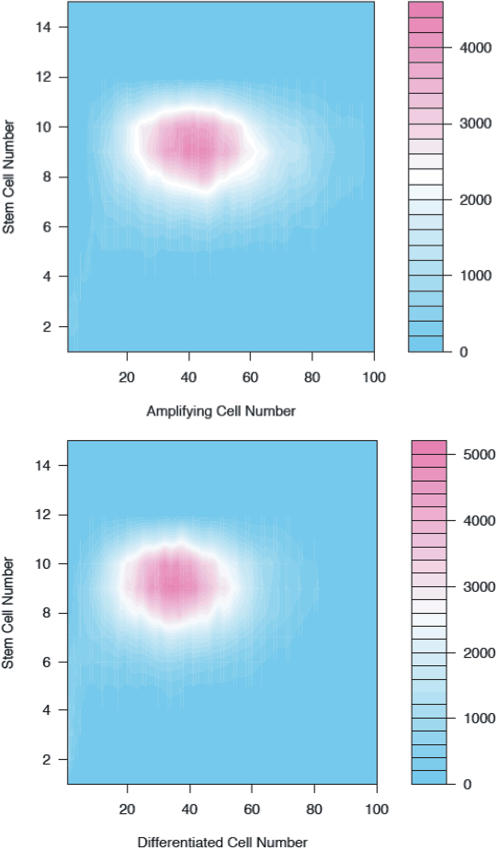
The results of the stochastic simulation can be shown as phase plots of stem cells and transit amplifying cells (uppere panel) or stem cells and fully differentiated cells (lower panel)

In Figure 3, we show the trajectories for transit amplifying (Figure 3, upper panel) and fully differentiated (Figure 3, lower panel) cells and the associated solution of the differential equation system. We see that the set of differential equations is a good proxy for the mean trajectory of the stochastic system and that the number of fully differentiated cells has a trajectory that mimics that of the transit amplifying cells, as it must. In Figure 4, we show the realizations of the stochastic system as phase plane plots of the density of stem cells and transit amplifying cells (upper panel) or stem cells and fully differentiated cells (lower panel). We see that a wide-range of cell numbers is expected due to the inherent stochasticity in the system.

**Figure 5:**
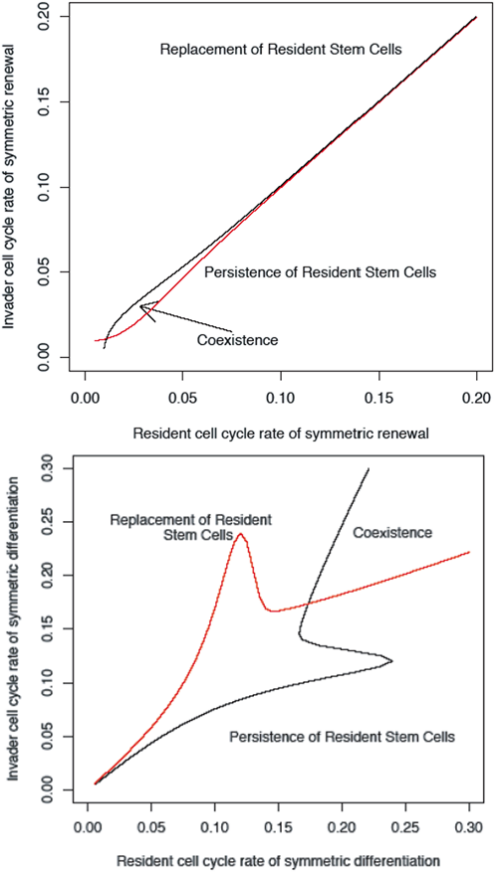
Deterministic replacement analysis allow us to determine when a mutation in one of the phenotypic parameters or the introduction of stem cells with different phenotypic parameters into a niche will lead to replacement of one stem cell clone by another, or coexistence of clones in the same niche. Upper panel) Stem cells differ in the cell cycle rate for symmetric renewal λ_1_. Lower panel) Stem cells differ in the cell cycle rate for symmetric differentiation λ_3_.

In Figure 5, we show two results of the invasion/replacement analysis. First (upper panel) we consider the invasion of a resident population of stem cells by a new phenotype with a different value of cell cycle rate for symmetric renewal 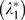. Here we see that in general if λ_1_ exceeds 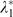 then the residents will resist the invasion by the new stem cell type and in general if the converse is true then the residents are excluded by the invading stem cell. However, there is a small region in which both kinds of stem cells coexist. Second (lower panel) we consider the replacement analysis when the value of the rate of the cell cycle for symmetric differentiation λ_3_ varies between the resident and invading stem cells. Here the region of coexistence is greater and the boundary curves, while still symmetrical are more complicated.

**Figure 6:**
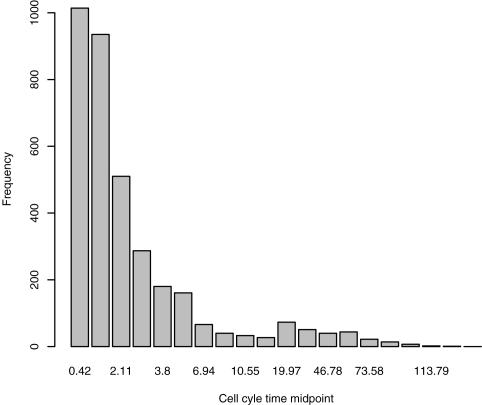
The forward simulation of the decisions by the focal stem cell allows us to generate the frequency distribution of the rates of transition, comparable to Figure 2.

In Figure 6 we show the frequency distributions of transitions by the focal stem cell following the behavior determined from the stochastic dynamic programming model. These data refer to 22,200 simulations in which there are about 3507 transitions of the simulated stem cells, of which only 6.42 per cent were symmetric renewal; the remaining transitions were asymmetric renewal. The minimum value of resources needed for a transition is *y_d_* = 5.5, but in the simulated stem cell population the average value of resources at the time of transition is 7.1, and the variance of those resources is 0.84. The average number of transit amplifying and background cells at the time of transition is 11.1, and the variance in those numbers is 53.5.

## Discussion

Phenotypic models of evolutionary processes have made important and long-lasting contributions in ecology and evolutionary biology [37]–[39] and in the study of cancer [20], [40]. They offer promise to help us understand great swaths of the life history and dynamics of stem cells and their descendants. Most of our work in this paper is in developing the population biology of stem, transit amplifying and fully differentiated cells in their niche to provide a fundamental structure for the analyses shown in Figures 5 and 6. This structure provides the framework for answering a wide range of questions about the evolutionary biology of stem cells. The results of Figure 5 show that it is possible to use evolutionary replacement analyses to understand consequences of manipulation of stem cell life history parameters *in vivo* and show, for example, that not all manipulations will be successful (the invading stem cell line cannot persist) or perhaps may be too successful (the invading stem cells fully replace the resident ones). We have not allowed mutations in the phenotypic parameters of the stem cell clones, but this is possible too with our methods [38] and would allow one to investigate how the clone changes over time due to mutation.

Figure 6 shows that it is possible to understand the key properties of stem cells – quiescence and great variability in activity – in terms of natural selection acting on the decisions of stem cells in response to the signals from the local microenvironment (the other stem cells) and from the more differentiated cells in the rest of the organism. Although it is comforting to see the similarity of Figure 2 and 6, one should remember that pattern does not imply process. However, the mechanistic model that underlies Figure 6 will allow us (elsewhere) to explore the key components of the process that lead to the pattern.

An empirical goal suggested by our work would be to more carefully identify these trade-offs and the feedback functions (described in detail in **Materials and Methods**) that characterize asymmetric renewal and symmetric differentiation. We have used simple exponential functions, but others – in which the all-or-nothing response is built in – are clearly possible [16]. How the strength of these signals will evolve also remains an open question.

Our work also provides a number of qualitative insights. For example, in healthy organisms, we should not expect the stem populations to be at their maximum sizes. Rather, the stochastic processes of birth and death will lead to population sizes smaller than the maximum. This, of course, makes identifying pathological situations more difficult, but should also help us avoid leaping to inappropriate conclusions. Furthermore, one cannot conceive of the transitions of stem cells (quiescence, symmetric renewal, asymmetric renewal, symmetric differentiation, apoptosis, or migration) in the absence of feedback from the amplifying transit cells and the fully differentiated cells. Indeed, they are crucial to what happens in the niche. We have also shown that a single framework can account for the observed ratio of fully differentiated to stem cells [41] by varying the phenotypic characteristics.

Many questions remain and there is much work to be done, ranging from the development of systems biology models for the phenotypic parameters, to the evolution of the signalling functions and their parameters, to the development of more complicated game theoretical aspects. Each will provide new insight into the evolutionary ecology of stem cells and their niches.

## Materials and Methods

### Another look at Till et al 1964

Till et al. [24] recognized that overdispersed data (in which the variance is much larger than the mean) such as theirs could not be described by a Poisson distribution. Instead, they fit a gamma distribution [25] to their data. The Poisson process is equivalent to the assumption that the probability of a stem cell being active in the next *dt* is λΔ*t*+*o*(Δ*t*), where *o*(Δ*t*) indicates times that are higher powers of Δ*t*. An improvement upon their approach is the following. It is known that there is variation in cell cycle times [42]. Thus, rather than fitting the counts with a gamma density, we assign a gamma density to λ, leading to a negative binomial distribution for the counts [25]. We use the method of moments to infer the parameters of this gamma density. Since the cell cycle time *T* = 1/λ, it is then an elementary calculation to determine the frequency distribution of cell cycle time, given the inferred gamma density.

### The stochastic kinetics of transitions

We use *s*, *a*, *d* for particular values of the number of stem, transit amplifying, or fully differentiated cells. We characterize the feedback network in Figure 1 as follows. We let Δ*S*, Δ*A* and Δ*D* denote the change in the number of stem, amplifying, or differentiated cells in the interval of time Δ*t*, which is assumed to be small. As noted before, *o*(Δ*t*) denotes terms that are higher powers of Δ*t* (typically Δ*t* ^2^, Δ*t* ^3^ etc.).

#### Symmetric Renewal



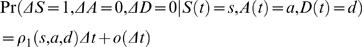
(4)

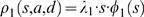
(5)


In this equation, λ_1_ = 1/*T*_1_ is the cell cycle time for stem cells that renew symmetrically. We assume no variation in this rate. The function Φ_1_(*s*) characterizes the inhibition of stem cells upon each other [43]. A variety of function forms are possible [16] but the key is that Φ_1_(*s*) decreases as *s* increases (which captures the inhibition); for computations we use Φ_1_(*s*) = *ln*(*K*)−*ln*(*s*).

#### Asymmetric Renewal



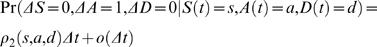
(6)

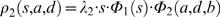
(7)In analogy to Eqn (5), λ_2_ is the cell cycle rate when a stem cell renews asymmetrically, with associated cell cycle time *T*_2_. Inhibition of symmetric renewal occurs for two reasons. First, negative feedback from stem cells on stem cells decreases overall activity. Second, negative feedback from existing transit amplifying and fully differentiated cells inhibits the production of transit amplifying cells. Thus Φ_2_(*a*,*d*,*b*) characterizes the signal from the transit amplifying and fully differentiated cells to the stem cells. Once again, this function will decrease as the number of transit amplifying and fully differentiated cells increases. For computations or analysis we use Φ_2_(*a*,*d*,*b*) = *e* ^−*s*(*a+d+b*)^ which is equivalent to a Hill function when the argument is small. ε measures the sensitivity of the stem cell population to what is happening in the population of transit amplifying and fully differentiated cells. Stem cells convert graded stimuli into all or nothing responses [44] and we have specifically chosen a graded rather than all or nothing response [16] because that allows the all or nothing response to emerge, rather than to be built into the model.

#### Symmetric Differentiation



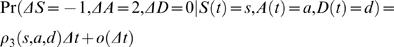
(8)

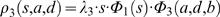
(9)Here λ_3_ and Φ_3_(*a*,*d*,*b*) are analogous to those above but can be understood as a ‘more desperate’ signal. For computations or analysis we use Φ_3_(*a*,*d*,*b*) = *e* ^−γ(*a*+*d*+*b*)^ where *γ* is the sensitivity parameter for symmetric differentiation.

#### Migration/Apoptosis

From the viewpoint of the focal stem cell, whether another stem cell undergoes apoptosis or migrates out of the niche (which is surely important for the state of the organism) is immaterial – the consequence is the same, a reduction in the number of stem cells in this niche. Thus we combine them in
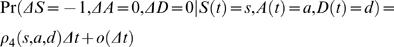
(10)

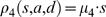
(11)Here μ_4_ measures the rate of migration of stem cells out of the niche plus the rate of apoptosis, both on a per stem cell basis.

#### Transit Cell Amplification

Transit cells amplify and since they send a signal back to the niche, their dynamics must be included. Hence
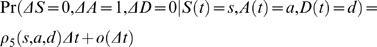
(12)

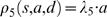
(13)Here λ_5_ is the cell cycle rate for transit amplifying cells. As mentioned above, we have compressed the *n* stages of development from transit amplifying to fully differentiated cell; unpacking this assumption can be done through a linear chain [38], [45], [46].

#### Transit Cell Mortality

Transit amplifying cells will disappear because of apoptosis (e.g. because they have too many DNA errors) and because they fully differentiate. We let μ_6_ denote the total per capita rate at which transit amplifying cells disappear; a fraction φ of them are converted to differentiated cells and the remainder experience apoptosis. Thus the mortality of transit amplifying cells is
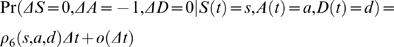
(14)

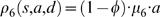
(15)


#### Transit Cell Differentiation

Transit amplifying cells that disappear but do not die are converted into fully differentiated cells as in
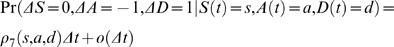
(16)

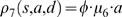
(17)Note that ρ_6_+ρ_7_ = μ_6_ *a,* the total rate at which transit amplifying cells disappear.

#### Mortality of Differentiated Cells

Finally, fully differentiated cells die, so that
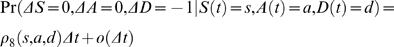
(18)

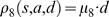
(19)Here μ_8_ accounts for all sources of mortality of fully differentiated cells.

### State dynamics using Gillespie's τ method

We use the Gillespie τ-method [47]–[50] to convert from rates of transitions in a small interval of time Δ*t* to the dynamics of the populations of cells. We assume that the time to the next transition of the system, τ, is a random variable with exponential distribution and mean time determined by the rate of all processes. The overall rate is
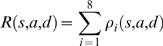
(20)so that
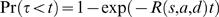
(21)Given the current values of *S*(*t*), *A*(*t*) and *D*(*t*) we first compute the time of the next transition by comparing the right hand side of Eqn 21 with a uniformly distributed random variable.

Next, given that a transition has occurred we compute
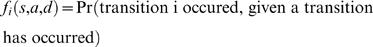
(22)Since when time τ has elapsed one of the transitions has occurred, we have
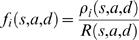
(23)Thus, the *f_i_* are the probability density function for the transitions of the system. It is also useful in numerical implementation to deal with the cumulative distribution function *F_i_*(*s*,*a*,*d*) defined by
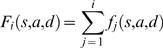
(24)


### Associated differential equations

The numbers of stem, transit amplifying, and fully differentiated cells are random variables. The rates of transitions of stem cells are nonlinear functions of the states, thus it is a nontrivial matter to connect the transition rates to ordinary differential equations for the states [51]–[56]. It is perhaps easiest to associate differential equations with the transitions if we interpret the differential equations as the mean of conditioned kinetics [56]. Thus, taking expectations and following the method of [56] we associate the following differential equations with the transitions described in the previous section
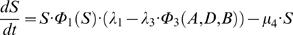
(25)

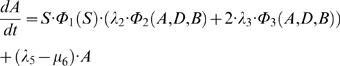
(26)

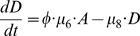
(27)This set of ordinary differential equations will allow us to develop a number of novel insights about stem cells, their niche, and replacement dynamics of stem cells in their niche.

### Biological constraints on parameter values

The transitions of stem, transit amplifying, and differentiated cells are characterized by a number of parameters. Biological realities will constrain some choices for parameter values. For example, we may anticipate that the probability that all stem cells in the niche either undergo apoptosis or migrate out of the niche is not too high since if it were the system could not persist. That is, if the niche empties (either through apoptosis or migration), it has essentially gone extinct. In computation, we choose parameters so that the probability of extinction of the niche is less than 20 percent.

Similarly, in most situations, the population of transit amplifying cells is much more active than the population of stem cells (with consequence that the number of amplifying cells will thus be much larger than the number of stem cells). For purposes of computation we use the condition ρ_5_>4ρ_1_.

We require that the number of support cells is sufficient such that when there is just one stem cell in the niche, the numbers of stem cells increase. In terms of the associated ordinary differential equations, this is equivalent to 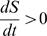 when *S* = 1. Since Φ_3_(*A*,*D*,*B*) will be a decreasing function of *A*, *D* and *B*, it follows from Eqn 25 that we require
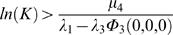
(28)In addition, we may expect there to exist links and trade-offs between the different parameters. For example, since symmetric renewal, asymmetric renewal and symmetric differentiation involve many of the same signals and processes, we may expect that the cell cycle rates λ_1_, λ_2_, and λ_3_ are correlated. Thus, for computations, we use λ_2_ = 2·λ_1_ and λ_3_ = 0.05·λ_2_. Similarly, cells that undergo high rates of replication are more likely to have errors in them and thus be more likely to undergo apoptosis. The simplest version of that trade-off is a linear one in which
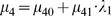
(29)

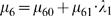
(30)For example, the disappearance of stem cells from the niche is decomposed into terms characterizing migration to other niches (μ_40_) and mortality due to apoptosis. The second equation above allows us to separate the disappearance of transit amplifying cells into those potentially converting to differentiated cells and those suffering apoptosis.

When transit amplifying cells disappear at a higher rate because of replication error, fewer of them will become fully differentiated cells. This can be captured by a trade-off between φ and λ_1_ as in
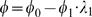
(31)In summary, we may think of the parameter set [λ_1_,λ_2_,λ_3_,μ_40_,μ_41_,λ_5_,μ_60_,μ_61_,φ_0_,φ_1_,μ_8_] as the phenotype of a stem cell and its descendants, or if all of the stem cells in a niche are identical descendants of a single cell, then these parameters are the phenotype of the stem cell clone. But, of course, all the stem cells in a niche need not be conspecifics. We thus turn to the competition between different stem cell phenotypes.

### Coexistence, replacement, and invasion

We can use evolutionary replacement analysis [38], [57]–[61] with the system of ordinary differential equations to explore the competition between and modification of stem cell clones and when manipulation of stem cell transition parameters will be successful. We now use *i* and *j* to index the stem cell clone with different phenotypic parameters, let γ*_ji_* denote the influence of transit amplifying and fully differentiated cells of type *j* on stem cell clone *i* so that
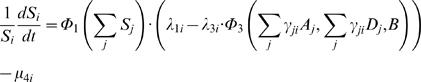
(32)

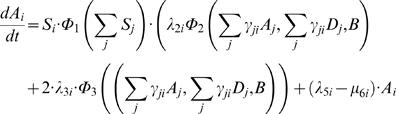
(33)

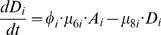
(34)


In these equations, we assume that all stem cells share the functional forms for the influence of transit amplifying and fully differentiated cells on asymmetric renewal and symmetric differentiation. After specifying the set of phenotypic parameters, we can integrate the coupled differential equations to determine the outcome of competition between any number of stem cell clones that differ in their phenotypic parameters.

However, to illustrate the ideas and explain them in as simple a manner as possible, consider the situation in which we wish to manipulate some of the phenotypic parameters in a niche by inserting a new stem cell with different parameters into an existing clone. In that case, we need to consider the stem cells present (the ‘resident’ stem cells) and the new one (the ‘invading’ stem cell). For definiteness, let us assume that the rate of symmetric differentiation of the residents is λ_3_ and of the invader is 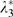. We first solve Eqns 25–27 for the steady state numbers of stem, transit amplifying, and fully differentiated cells with the resident phenotype which we denote by 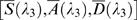 to focus our attention on these steady state values as they depend upon the value of λ_3_ (clearly, they depend upon the other phenotypic parameters too, but we only consider the manipulation of λ_3_ here).

To make the notation simpler let us return to Eqn 25 and rewrite it as
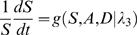
(35)Then 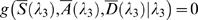 since those values correspond to a steady state. Now imagine that we introduce a stem cell with different phenotypic parameters. We let 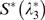 denote the number of stem cells with symmetric differentiation rate 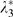. A number of outcomes are possible.

First, it is possible that 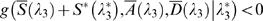 when 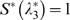 (initially there are no transit amplifying or fully differentiated cells descended from the invading stem cells). In this case, the number of stem cells with symmetric differentiation rate 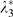 declines and we say that the resident stem cells resist invasion or replacement.

Second, it is possible that 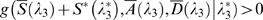 when 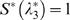. In this case, the number of invading stem cells will increase, and lead to transit amplifying and fully differentiated descendants. One effect will be that the resident stem cells will be disturbed from their steady state. Then their numbers may decline, in which case we say that the invading stem cell has replaced or excluded the resident stem cells. Alternatively, after the perturbation by the invading them cell (and the production of transit amplifying and fully differentiated cells from the invader) the resident stem cells may remain steady or even increase. In that case, we say that the two kinds of stem cells coexist.

### The focal stem cell in its niche

We now describe how one may characterize the behavior of a focal stem cell in its niche. To be sure, thinking of a single stem cell having behavior while others cells in the niche (and the transit amplifying and fully differentiated cells) have a fixed behavior is a simplification. Ultimately, one should view stem cells in their niche as playing a dynamic cooperative game, but we reserve that for subsequent work. For the time being, we focus on the state dependent life history of a focal stem cell, determining the optimal life history through stochastic dynamic programming [33]–[36].

Like all cells, the focal stem cell must accumulate resources for division. We denote by *Y*(*t*) the resources available for cell division at time *t*, assuming that a minimum resource level *y_d_*. We assume that the inhibition of the focal stem cell by other stem cells also affects the flow of resources to the focal stem cell and has the same functional form as used in the inhibition of cell cycling. Thus
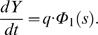
(36)Here *q* can be scaled out by interpreting *t* as multiples of 1/*q*, so we set it equal to 1.

We assume that the organism gains fitness from these fully differentiated cells at rate *R_F_*(*d*) when there are *d* fully differentiated cells sending signals to the focal stem cell. We assume that the decision of the focal stem cell is made at the time of the next transition of the stem, amplifying or differentiated cells. Using the methods describe above, we compute τ(*s*,*a*,*d*), which is the random variable describing the time to the next transition, and the vector Δ = (Δ*S*,Δ*A*,Δ*D*) that describes the changes in stem, transit amplifying and differentiated cells when a transition occurs.

When this transition occurs, the focal stem cell has level of resources *y*. If *y* is less than the resources available for differentiation, then the focal stem cell cannot do anything other than remain quiescent. In that case
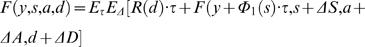
(37)which we define as *V*_0_, the fitness value of remaining quiescent. The right hand side of this equation represents the future accumulation of fitness and is averaged over the time of the next transition and the change in the cell population when that transition occurs.

If *y* exceeds *y_d_*, then in the simplest case the focal stem cell may remain quiescent (with value *V*_0_ given above), renew symmetrically (with value *V*_1_ to be determined) or renew asymmetrically (with value *V*_2_ also to be determined). Allowing for full differentiation of the focal stem cell, or migration out of the niche requires certain complications which we will postpone for a later paper. After a division resources *y*−*y_d_* remain for correcting errors in the daughter cells. We thus let Ψ(*y*−*y_d_*) denote the probability that a daughter cell is error free (i.e. does not undergo apoptosis), given that *y*−*y_d_* resources are used for correcting errors. For computations we use Ψ(*z*) = 1−exp(−γ*_c_z*) where γ*_c_* is a parameter.

If the stem cell renews symmetrically, we assume that the focal stem cell remains error-free and a daughter stem cell joins the other stem cells in the niche. Assuming segregation of errors upon division [29], [31], all resources remaining after division are used to ensure that the daughter stem cell is error free. We thus have
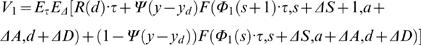
(38)On the other hand, if the stem cell renews asymmetrically, a new transit amplifying cell is produced and the resources remaining after division are used to correct errors in the new transit amplifying cell. In this case
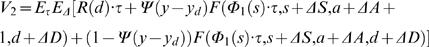
(39)Thus, when *y* exceeds *y_d_* we have
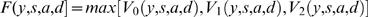
(40)Note that there is no explicit time in any of these equations. Thus, they cannot be solved by the usual method of backward iteration [33]–[36]. Rather, we determine *F*(*y*,*s*,*a*,*d*) by value iteration (see [62] for a general discussion and [63] for an application in behavioral biology). When the value iteration has stabilized, we have found in addition to fitness, the optimal decisions *i* ^*^(*y*,*s*,*a*,*d*) of the focal stem cell when its accumulated resources for division are *y*, there are *s* other stem cells in the niche and the number of transit amplifying and differentiated cells sending signals to the niche are *a* and *d* respectively. We then use the Markov simulation described previously to create the environment of the focal stem cell and follow its subsequent behavior (quiescence, symmetric renewal, or asymmetrical renewal) as the number of stem cells in the niche and the number of amplifying and differentiated cells change through the stochastic processes described previously. In this way, we are able to construct the times between transitions of the focal stem cell, and thus the analogue of the distribution of Till et al. [24].
